# Microbial Enzymes and Their Applications in Industries and Medicine 2016

**DOI:** 10.1155/2017/2195808

**Published:** 2017-03-28

**Authors:** Periasamy Anbu, Subash C. B. Gopinath, Bidur Prasad Chaulagain, Thangavel Lakshmipriya

**Affiliations:** ^1^Department of Biological Engineering, College of Engineering, Inha University, Incheon 402-751, Republic of Korea; ^2^Institute of Nano Electronic Engineering (INEE), Universiti Malaysia Perlis, 01000 Kangar, Perlis, Malaysia; ^3^School of Bioprocess Engineering, Universiti Malaysia Perlis, 02600 Arau, Perlis, Malaysia; ^4^Directorate of Research and Training, Himalayan College of Agricultural Sciences and Technology (HICAST), P.O. Box 25535, Kalanki, Kathmandu, Nepal

Enzymes are biocatalysts that play an important role in metabolic and biochemical reactions [1]. Microorganisms are the primary source of enzymes, because they are cultured in large quantities in short span of time and genetic manipulations can be done on bacterial cells to enhance the enzyme production [2–4]. In addition, the microbial enzymes have been paid more attention due to their active and stable nature than enzymes from plant and animal [2–4]. Most of the microorganisms are unable to grow and produce enzyme under harsh environments that cause toxicity to microorganisms. However, some microorganisms have undergone various adaptations enabling them to grow and produce enzymes under harsh conditions [5, 6]. Recently several lines of study have been initiated to isolate new bacterial and fungal strains from harsh environments such as extreme pH, temperature, salinity, heavy metal, and organic solvent for the production of different enzymes having the properties to yield higher [6–9]. This special issue covers six articles including one review article, highlighting the importance and applications of biotechnologically and industrially valuable microbial enzymes.

There are redundancies in genetic code that amino acid might be encoded by multiple synonymous codons. This scenario gives an opportunity to choose a codon other than the naturally occurring one in the genome to optimize the production with heterologous expression system. Codon optimization in another sense is a guided mutagenesis in the gene expression system which can be utilized for the benefits of human kind, ranging from industrial agriculture to medicine. J. Wang et al. have applied a series of strategies to improve the expression level of recombinant endo-*β*-1,4-xylanase from* Aspergillus usamii* in* Pichia pastoris*. The endo-*β*-1,4-xylanase gene (xynB) from* A. usamii *was optimized for expression in* P. pastoris*. Their analysis showed the codon for amino acid residues in* P. pastoris* is different from the original codon of* A. usamii. *Thus they replaced the codons in endo-*β*-1,4-xylanase gene to fit to the cellular environment of* P. pastoris*. Similarly they optimized the codons of* Vitreoscilla hemoglobin gene *(vhb) to fit to heterologous expression system in* P. pastoris* cell system. While optimizing codons they replaced the AT-rich stretches with GC-rich stretches, because G+C content affects the secondary structure of mRNA and influences the expression level of heterologous gene. Totally, 105 and 57 amino acids were optimized in native xynB and vhb, respectively. Besides optimizing the genetic system, J. Wang et al. have also optimized the environment to express those recombinant genes. The codon optimized* vhb* gene has significantly improved the oxygen availability for host* P. pastoris* since oxygen supply is one of most critical factors for the cell growth and heterologous protein expression in recombinant* P. pastoris*. By optimizing the temperature effect on the system they increased the xylanase production combined with higher cell viability. Overall, this work has supported the notion of genetic engineering in code optimization and a piece of novel work for the recombinant biotechnology.

X. Jia and his colleagues have produced the recombinant catalase in* Escherichia coli *from* Geobacillus *sp. gene* (Kat)*. This* Kat *gene has 1,467 bases and encoded a catalase with 488 amino acid residuals with 81% similarity to the previously studied* Bacillus *sp. catalase. Fermentation broth of the recombinant* E. coli *showed a high catalase activity level up to 35,831 U/mL. The purified recombinant catalase had a specific activity of 40,526 U/mg and a* Km *of 51.1 mM. The optimal reaction temperature of this recombinant enzyme is 60 to 70°C, and it exhibits a high activity over a wide range of reaction temperatures. The enzyme retained 94.7% of its residual activity after incubation at 60°C for 1 hour. The high yield and excellent thermophilic properties of this recombinant catalase have valuable features for industrial applications.

In the work performed by M. Karkovska et al., a laboratory column-type bioreactor for removing a toxic D-lactate on permeabilized thermotolerant methylotrophic yeast (*Hansenula polymorpha *“tr6”) cells and alginate gel was constructed and tested. Using recombinant* H. polymorpha *“tr6” overproduced the D-lactate: cytochrome c-oxidoreductase. At about 6-fold overexpression of D-lactate, cytochrome c-oxidoreductase under a strong constitutive promoter* (prAOX)* was demonstrated. Overexpression of D-lactate dehydrogenase coupled with the deletion of L-lactate-cytochrome* c *oxidoreductase activity opens the possibility for usage of the strain as a base for construction of bioreactor for removing D-lactate from fermented products due to the oxidation to nontoxic pyruvate.

RNases are regarded as alternative to classical chemotherapeutic agents due to their selective cytotoxicity towards tumor cells. In the work demonstrated by Y. Sokurenko et al., extracellular ribonuclease from* Bacillus licheniformis *(balifase), a new member of the N1/T1 RNase superfamily, is shown to have antitumor effects. The new RNase produced is with a high degree of structural similarity with binase (73%) and barnase (74%) having a molecular weight of 12422 Daltons and pI 8.9. The physicochemical properties of balifase are similar to those of barnase. The gene organization and promoter activity of balifase are closer to binase. In this study, similar to the biosynthesis of binase, balifase synthesis was induced under phosphate starvation; however, in contrast to binase, balifase does not form dimers under natural conditions. This study also proposed that the highest stability of balifase allows retaining its structure without oligomerization.

In their review, S. Gopinath et al. focused on importance of microbial amylase in the field of biotechnology. In this article, the authors have discussed isolation and screening of amylase from bacterial strains. They also discussed the improvement of enzyme production by optimization and recombinant DNA technology. The major advantages of microbial enzymes in many industries such as food, detergent, pharmaceutical, paper, and textile industries are discussed. The technologies of high-throughput screening and processing with efficient microbial species, along with the ultimate coupling of genetic engineering of amylase-producing strains, will all help in enhancing amylase production for industrial and medicinal applications.

Efficient delivery of drug to the target cell is very important for treatment. Drugs with brain tissue related treatments are hindered by the blood brain barrier. Flurbiprofen is one of the potent drugs that may help to prevent Alzheimer's disease but it has problem with blood-brain barrier permeability. To overcome this problem J. Xin et al. have worked to design and modify this drug as L-ascorbyl flurbiprofenate with the addition of ascorbic acid as a specific carrier system for brain delivery. In the process they have tried to optimize lipase-catalyzed esterification and transesterification. They found that synthesis of L-ascorbyl flurbiprofenate was influenced by the specific reaction conditions and the most important step was the efficient removal of byproducts during the reaction. While comparing those esterification and transesterification methods, although the rate of esterification was found slower than that of transesterification, from the standpoint of productivity and the amount of steps required, lipase-catalyzed esterification was found superior for the synthesis of L-ascorbyl flurbiprofenate.

*Periasamy Anbu* *Subash C. B. Gopinath* *Bidur Prasad Chaulagain* *Thangavel Lakshmipriya*
